# An Interprofessional Primary Palliative Care Curriculum for Health Care Trainees and Practicing Clinicians

**DOI:** 10.1089/pmr.2021.0074

**Published:** 2022-05-05

**Authors:** Brook A. Calton, Naomi Saks, Thomas Reid, Nancy Shepard-Lopez, Bridget Sumser

**Affiliations:** ^1^ Division of Palliative Medicine and Geriatrics, Department of Medicine, Massachusetts General Hospital, Boston, Massachusetts, USA.; ^2^ Division of Palliative Medicine, Department of Medicine, The University of California, San Francisco, San Francisco, California, USA.

**Keywords:** curriculum development, interprofessional education, primary palliative care education

## Abstract

**Background:**

Equipping all interprofessional clinicians with foundational palliative care competencies is essential to address the complex needs of the growing number of adults living with chronic, progressive, or life-threatening serious illness. There is a paucity of high-quality, open-access primary palliative care curricula and to the best of our knowledge, none designed interprofessionally.

**Objective:**

As an interprofessional team, we aimed at designing and evaluating an interactive primary palliative care education curriculum for interprofessional clinicians and trainees.

**Design:**

We developed a curriculum that includes nine 55-minute interactive modules facilitated by two interprofessional clinicians in small groups of 8–12 interprofessional learners.

**Setting/Subjects:**

Thirty-two practicing interprofessional clinicians from the San Francisco Bay Area enrolled in the pilot.

**Measurements:**

Pilot curriculum evaluation included electronic surveys pre- and post-module and at completion of the full curriculum.

**Results:**

The final evaluation response rate was 44%. Ninety-three percent of survey respondents rated the curriculum's quality as “very good” or “excellent”; 86% of respondents felt the curriculum was “extremely” or “very useful” to their clinical practice. Comparing pre- and post-module survey data, statistically significant (*p* < 0.01) improvements in learner confidence were seen for each of the 25 curriculum learning objectives with an average improvement of 2.8 points.

**Conclusions:**

The curriculum was well received and was associated with an increase in learner confidence. This novel, flexible, and tuition-free curriculum fills an important educational gap and can be used to equip frontline, interprofessional clinicians with the core palliative care knowledge, skills, and attitudes to take the best possible care of seriously ill patients and families.

## Introduction

A well-documented gap exists between the number of seriously ill Americans who would benefit from high-quality palliative care and the number of subspecialty clinicians equipped to deliver it.^1^ A critical strategy to address the lack of specialty clinicians is to increase capacity and access to care for the seriously ill through primary palliative care education for non-palliative care clinicians.^2,3^

Primary care and other health care professionals who have continuity with patients are well positioned to integrate palliative care alongside preventive and curative care into their established practices.^4^ The Institute of Medicine states that all professions require additional training and skills in primary palliative care.^5^

Similar to specialty palliative care, primary palliative care is best delivered by a collaborative team of interprofessional clinicians.^4^ Providing team-based serious illness care is a skill that must be learned, practiced, and is not consistently taught.^5^ Interprofessional education (IPE), defined by the World Health Organization as, “occur[ing] when two or more professionals learn about, from and with each other,”^6^ can prepare clinicians to provide effective team-based care and improve quality outcomes and patient safety.^7^

IPE can support learners in incorporating new perspectives, negotiating differences in opinion, understanding roles, and improving communication and collaboration.^8^ It is widely accepted that curricular innovations in primary and palliative care should routinely include IPE.^5^

Although primary palliative care educational offerings are increasingly prevalent, they vary widely in intended audience, scope, and cost.^2^ Based on our environmental scan, most primary palliative care offerings currently available are brief,^9,10^ asynchronous,^9–14^ fee-based,^12,13^ and/or are designed by and for a single profession.^14,15^ Longitudinal, interactive, and comprehensive curricula designed by a team of interprofessional clinicians for interprofessional clinicians remain relatively uncommon.^15^ While some high-quality, interactive, and interprofessional curricula including a continuing medical education course “Practice-PC” offered through the University of California, San Francisco^16^ and palliative care certificate programs through the University of Washington and Colorado University exist,^17,18^ these require significant time (i.e., year-long) and financial commitments (ranging between $5,500 and $26,100).

There are multiple reasons as to why the interprofessional primary palliative care curriculum is challenging to develop and remains infrequent. Developing training grounded in universal standards of practice can prove difficult, because: (1) Educators are still in the process of delineating between specialty and primary palliative care^19^; (2) Some (social work and nursing) but not all disciplines have defined discipline-specific primary palliative care competencies^20,21^; and (3) To the best of our knowledge, interprofessional competencies do not exist. The quality of and access to in-person, live teleconference, or online offerings varies greatly depending on learner interest, time constraints, professional reimbursement, geography, and faculty availability. Logistical challenges, scheduling constraints, professional power differences, territorial dynamics, and limited funding are also obstacles to IPE.^7^

To address the educational gaps discussed earlier, we developed a nine-hour interprofessionally informed and delivered curriculum to prepare interprofessional, graduate medical education trainees and practicing clinicians on the front lines of caring for patients and families with serious illness with the knowledge, skills, and attitudes to practice primary palliative care. The purpose of this article is to describe our interprofessional curriculum development process, exploratory outcomes from our pilot with 32 interprofessional clinicians, lessons learned, and disseminate the curriculum for use by outside institutions.

## Materials and Methods

### Curriculum development

Our curriculum development team included a social worker, chaplain, nurse practitioner, and two board-certified Hospice and Palliative Medicine physicians, all practicing specialty palliative care at an urban academic medical center. All team members had practiced specialty palliative care for between 7 and 15 years and previously served as faculty in designing and facilitating interprofessional palliative care education.

Within our first two in-person curriculum development meetings, we defined two beliefs that all team members agreed would form our curriculum's foundation and guide upcoming work: (1) All clinicians of all disciplines working with people who are living with serious illness can benefit from primary palliative care education; and (2) Because palliative care should be provided through interprofessional teams, interprofessionally created and facilitated education experiences emulate the most authentic experience of teaching, learning, and clinical practice.

We employed in-person brainstorming and group discussion among all interprofessional team members to craft three global curriculum learning objectives, which are below.

On completion of the curriculum, learners should be able to:

1. Recognize that all interprofessional clinicians are able to participate in the palliative care philosophy and practice across health care settings.
2. Identify patient needs and preliminary interventions in the eight National Consensus Project (NCP) palliative care domains.^4^
3. Demonstrate understanding of the individual and interprofessional team's role in primary palliative care from time of diagnosis to end of life and bereavement.

Through the collective expertise of the curriculum development team and review of the NCP guidelines,^15^ we identified eight curriculum modules. Ultimately, we created a ninth module, separating Psycho-social care and Spiritual and Cultural Care into distinct modules based on learner feedback. Two curriculum development team members from different disciplines were assigned to each module. The two assigned team members were responsible for the initial draft of the module's learning objectives, informed by clinical expertise and the NCP guidelines.

The module's draft learning objectives were reviewed and edited through a series of in-person discussions with the larger curriculum development team to ensure an interprofessional perspective (Table 1). The two curriculum development team members responsible for the module then created a draft of the module in Microsoft PowerPoint and accompanying facilitator guides. The full curriculum development team met to review each model together and provide comments; this process of ongoing feedback and revisions continued until we reached consensus across all team members. We performed 29 hours of feasibility testing of select modules that were a work-in-progress with volunteer groups of interprofessional learners.

**Table 1.** Learning Objectives by Module

Module number	Module name	Module learning objectives
1	Introduction to palliative care	• Define primary vs. specialty palliative care • Describe common serious illness trajectories • Differentiate between patients who would benefit from palliative care and those who would benefit from hospice
2	Psychosocial care	• Appreciate how the psychological and social aspects of patients' lives influence their experience of serious illness • Learn simple screening methods to identify psychosocial needs • Understand how to provide basic support and other resources to address these needs
3	Spiritual and cultural care	• Appreciate the importance of the cultural and spiritual domains of palliative care • Become familiar with screening patients to identify cultural and spiritual needs and strengths • Understand how to provide basic support and other resources to address need
4	Serious illness communication (Part 1)	• Describe four skills to enhance your communication with seriously ill patients and families • List best practices when communicating with seriously ill patients and families
5	Serious illness communication (Part 2)	• Describe how capacities impact your communication with seriously ill patients • Demonstrate four skills to enhance your communication with seriously ill patients
6	Pain management	• Discuss how a patient's biologic/psychosocial/spiritual/cultural identity informs their experience of pain • Identify evidence-based pain assessment tools • Describe a multi-modal approach to managing pain
7	Symptom management	• Appreciate the frequency with which seriously ill patients experience symptoms Demonstrate a holistic approach to symptom management • Identify key assessment and management strategies for three of the most common symptoms
8	Advance care planning	• Appreciate the frequency with which seriously ill patients experience symptoms • Demonstrate a holistic approach to symptom management • Identify key assessment and management strategies for three of the most common symptoms
9	Care at the end of life	• Describe what patients/families want at the end of life • Identify common signs and symptoms in final days of life • Recognize religious/spiritual/cultural practices and rituals before/after death • Name strategies to identify and address grief and bereavement needs • Recognize approaches to identifying professional grief

### Curriculum overview

The final curriculum includes nine 55-minute modules (Table 1). Each module consists of a PowerPoint slide deck with a companion facilitator guide. The modules are designed to elicit group participation and discussion. Each module features group activities, case discussion, and reflection and therefore is best suited to an interactive small group experience in-person or virtually (not for asynchronous use). Modules can be free-standing or offered as a series. They can be adapted for clinicians of various roles, training, specialties, and experience levels, including single profession and mixed interprofessional groups.

We designed the modules to be taught by a pair of clinicians from different professions who are experts in palliative care and/or have significant clinical experience caring for patients with serious illness. The modules are designed to be delivered to small groups of 8–12 graduate medical education trainees and/or practicing clinicians from different disciplines.

### Curriculum implementation

We piloted the full initial curriculum (consisting of eight modules where Psycho-Social-Spiritual-Cultural Care was one module) with three interprofessional clinician small groups (Table 2). Pilot group participation was voluntarily (we selected teams based on previously expressed interest in palliative care training). The participants for each small group were determined by that group's team leader (i.e., Medical Director of the ALS Clinic), and participation was mandatory.

**Table 2.** Distribution of Interprofessional Clinicians by Small Group

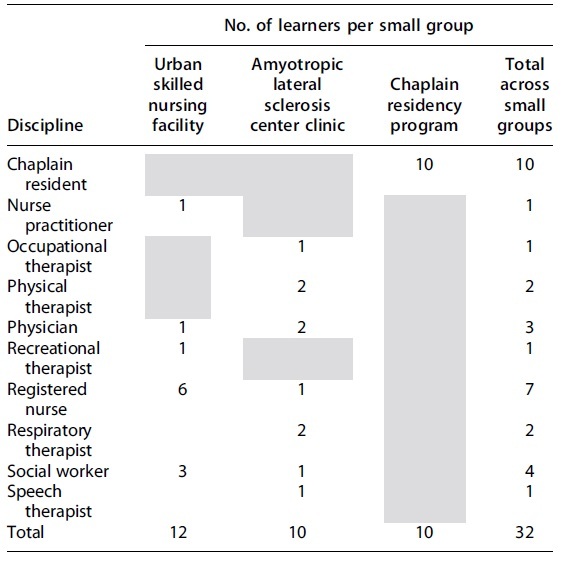

Gray shading signifies zero.

Teaching faculty were members of the curriculum development team. When teaching a single professional group, we aimed at having that profession represented in the teaching team (i.e., palliative care chaplain co-teaching with another facilitator for the group of Chaplains Residents), though all modules can be taught by any combination of professions (with the exception of Pain Management and Symptom Management that requires one of the facilitators to be either a physician or a nurse). Coupling facilitators from different professions modeled interprofessional practice and created shared experiences to inform curricular edits.

The number of modules delivered in one sitting varied (1–4), depending on learner and faculty availability and needs. The goal was to complete each curriculum pilot within 8 weeks (actual range 4–10 weeks). There was no prerequisite knowledge for target learners, though all had significant clinical contact with seriously ill patients and families.

At the end of each of the three pilot curriculum runs, we reviewed participant feedback by using our survey instrument, informal participant feedback verbally and by email, and teaching faculty observations by using a structured written format. The interprofessional curriculum development team edited the curriculum between pilot groups to improve it. All team members approved the updated version of each module before the next pilot group.

### Curriculum evaluation

This project was deemed exempt by the UCSF Institutional Review Board. Qualtrics was used for online survey administration and Microsoft Excel was used calculate descriptive statistics and frequencies.

Learners were sent a pre-module survey (Supplementary Appendix SA1) one week before their small group session (1–4 modules depending on scheduling). For each of the module's learning objectives, the survey asked learners to rate their confidence on a scale of 1 (“Not at all confident”) to 10 (“Completely confident”).

Learners were asked to fill out their post-module survey (Supplementary Appendix SA1) within two weeks of completing the corresponding module(s), which included the same questions as the pre-module survey and asked learners to what extent they agreed (on a 6-point bipolar scale, strongly disagree to strongly agree) that the cases/examples used in the module were relevant, the module reflected an interprofessional viewpoint, and the module was interactive.

We categorized learners who selected “agree” or “strongly agree” as “agree” for this analysis. The survey asked learners to report whether the time for the module was appropriate on a 5-point Likert scale from “far too little” to “far too much.” “Learners who selected ‘too little’ or ‘far too little’ were combined into one category-called ‘too little’—for this analysis.” Finally, we asked for learner comments on the qualitative strengths and weaknesses of each module.

On completion of the full course, learners were given two weeks to complete their final course evaluation (Supplementary Appendix SA2). The evaluation inquired about the curriculum's overall quality and applicability, collected a Net Promoter Score (NPS),^22^ and asked for global course comments. For this project, the NPS was used to determine how likely participants were to recommend the course to a colleague. The NPS has a range of −100 to 100, with a score >50 considered “excellent.”^22^

## Results

A total of 32 interprofessional clinicians (Table 2) participated in the curriculum pilot.

Pre-module completion rates ranged from 64% to 94%; post-module completion rates were 47%–88%. Learner confidence improved statistically significantly (*p* < 0.01) for each of the 25 course learning objectives, with an average improvement of 2.8 points in learner confidence. For each module, respondents agreed that the cases and examples were relevant to their clinical practice, reflected an interprofessional viewpoint, and were interactive (Table 3). Most respondents felt the time allotted for each of the module was “just right” though more than one-quarter of respondents felt there was “too little” time for the Psycho-Social-Spiritual-Cultural Care (32%), Serious Illness Communication (Part 1) (33%), and Advance Care Planning (31%) modules.

**Table 3.** Module-Specific Evaluation

Module no. and title	The cases and examples used in this module were relevant to my practice, % agreed^a^ (*n*/*N*)	This module's content reflected an interprofessional viewpoint, % agreed^a^ (*n*/*N*)	The presentation of this module was appropriately interactive, % agreed^a^ (*n*/*N*)
1: Introduction to PC	79 (22/28)	86 (24/28)	89 (24/27)
2: Psycho-social-spiritual-cultural care	86 (24/28)	86 (24/28)	86 (24/28)
3: Communication 1	94 (17/18)	88 (14/16)	94 (17/18)
4: Communication 2	100 (14/14)	86 (12/14)	93 (13/14)
5: Pain management	77 (17/22)	91 (20/22)	91 (20/22)
6: Symptom management	81 (17/21)	95 (20/21)	95 (20/21)
7: Advance care planning	88 (14/16)	94 (15/16)	94 (15/16)
8: Care near the end of life	95 (19/20)	95 (19/20)	95 (19/20)

^a^ Percentage agreed includes participants who selected “strongly agree” or “agree” on a 6-point Likert scale.

The final course evaluation response rate was 44% (14 of 32 participants). Thirty-six percent (*n* = 5) of survey respondents rated the overall quality of the course as “excellent”; 57% (*n* = 8) rated it as “very good.” Twelve of 14 respondents rated the overall course as “extremely” (*n* = 4) or “very” (*n* = 8) useful to their clinical practice. The calculated NPS for the course was 64. One respondent commented, “The program was practical and understandable. The concepts learned after each module could be immediately implemented on my unit.” Another stated, “I would love to have our group take this course every couple of years as new team members join and others leave and just as a refresher for those who took this course.”

## Discussion

The goal of this curriculum is to equip interprofessional graduate-level trainees and practicing clinicians with foundational primary palliative care knowledge, skills, and attitudes to provide the best possible care for seriously ill patients and families. In our exploratory analysis, learners reported significant improvements in their confidence across all 25 course learning objectives. Participants agreed the curriculum was relevant, interactive, and represented an interprofessional viewpoint; the vast majority would recommend the curriculum to others.

This curriculum adds to the primary palliative care education landscape by virtue of it being: (1) interactive; (2) of moderate time commitment (nine hours); (3) designed for interprofessional clinicians by interprofessional clinicians; and (4) available online without a fee for clinicians to incorporate for primary palliative care interprofessional training at their own institutions. For clinicians who choose to build their own interprofessional curricula, we offer lessons learned below based on our experience to inform your efforts.

### What we learned

Lessons learned include an appreciation for the sheer amount of time, energy, and thought it takes to build an interprofessional curriculum and reach group consensus. Future curriculum development teams would benefit from dedicated attention early on to group ground rules, expectation and role setting, and mission/vision.

Reflecting on our teaching experience, we believe it was essential for this curriculum to be taught by two facilitators from two different professions, preferably one medical and one non-medical (i.e., physician/social worker [MD/SW], nurse practitioner [NP]/chaplain not MD/NP). We felt strongly that these pairings enhanced the curriculum by offering different medical and psychosocial perspectives simultaneously. There may also have been value for learners in observing two facilitators from different disciplines model working together and navigating disagreements when they arise (aka “the hidden curriculum”). Our experience is supported by the literature demonstrating that interprofessional teaching can improve a learner's ability to recognize bias, think critically, tolerate ambiguity, and appreciate ethical considerations.^8^

One challenge of IPE we identified is the tension between keeping curriculum materials general enough, so that they are useful to learners across specialties (i.e., cardiology and surgery), settings (i.e., inpatient and outpatient), and professions (i.e., nursing and social work). It is neither realistic nor in the spirit of the curriculum to develop new modules tailored to the specific needs of each learner group. In the facilitator notes, we have recommended that if a patient case does not fit the needs of the group, the facilitator should brainstorm a new case to substitute.

The time required to run the full curriculum may challenge implementation and sustainability. We estimate that 25 hours of time/facilitator is required: 9 hours of prep (1 h/module), 9 hours of teaching (1 h/module), and 7 hours of faculty coordination to teach the full course. We found that presenting no more than 2 modules at a time was ideal to optimize adult learner enthusiasm and engagement. Arranging for protected learner time is an eternal challenge.

For our pilot, we met one-on-one with administrative leaders of each learner group to understand the prospective participants' needs and clinical schedules. When creating the schedules for each learner group, we prioritized scheduling the teaching sessions at times when the fewest number of participants had clinical responsibilities. Learner group-specific mechanisms were in place in circumstances where a learner needed to be released from their clinical duties and cross-coverage was arranged.

Protected faculty time using institutional or grant funding support or clinical productivity credit to prepare and teach the course is recommended to ensure the curriculum's sustainability. Depending on faculty bandwidth and learner needs, the entire curriculum does not need to be taught. The modules are free-standing and can be offered individually (except the two communication modules).

### Limitations

Although curriculum feedback was positive, evaluation was limited by our survey instrument and lower than expected survey completion for some modules. A higher response rate may be achievable by incentivizing learners to complete the electronic surveys, or have the learners complete the survey before leaving the teaching sessions. Self-reported learner confidence does not necessarily translate to learner competence.^23^

In addition, measuring the impact over time (i.e., with a follow-up survey one to six months out from curriculum completion) would help quantify the curriculum's impact on real-life clinical practice and retention of curricular concepts. Although we are presenting summary data across the pilot groups, we did make small edits to the curriculum between pilots, which may make this summary data less accurate.

### Future directions

Future directions include a more robust curriculum assessment. Focus groups with learners to understand their experience learning in an interprofessional group and from interprofessional teaching teams are also warranted. We are providing facilitator training to the ∼40 interprofessional palliative care clinicians at our academic medical center. The full curriculum is available online (Supplementary Curriculum Link), including facilitator training guides for clinicians to implement at their institution for interprofessional primary palliative care training. We welcome your feedback.

## Supplementary Material

Supplemental data

Supplemental data

Supplemental data

## Supplementary Material

Supplementary Appendix SA1

Supplementary Appendix SA2

Supplementary Curriculum Link

## Abbreviations Used

IPE: interprofessional education
NCP: National Consensus Project
NPS: Net Promoter Score
