# Supplementary material for: An Interprofessional Primary Palliative Care Curriculum for Health Care Trainees and Practicing Clinicians
Source: Palliat Med Rep. 2022 May 5;3(1):80–6. doi: 10.1089/pmr.2021.0074 (PMC9153988; doi:10.1089/pmr.2021.0074)
Supplement: Supplemental data [file Suppl_AppendixSA1.pdf]

Primary Palliative Care Curriculum Pre-Post Module Surveys

<u>Pre-Module Survey(s)</u>

Send out one week before the modules you plan to teach. AKA If you are teaching modules 1 and 2, send out the pre-survey for modules 1 and 2 one week before you teach. Include the following into with each survey:

Dear [PARTICIPANT NAME]:

You have enrolled in a primary palliative care education course along with your group: [INSERT GROUP NAME]. The following is a brief self-assessment covering the modules you have been enrolled to take, which are listed on the next page.

If you have any questions about the course, please contact [INSERT EMAIL].

We look forward to seeing you soon!

<u>Module 1: Introduction to Palliative Care</u>

On a scale of 1-10, how *confident* are you in your ability to perform each of the following learning objectives?

	Not at all confident Completely Confident<br>1 2 3 4 5 6 7 8 9 10
Introduce palliative care to a patient or family member ()	
Differentiate between patients that would benefit from palliative care and those who would benefit from hospice ()	
Define serious illness ()	
Identify 2 principles and practices of palliative care ()	

Property of UC Regents, B. Calton, B. Sumser, N. Saks, T. Reid, N. Shepard-Lopez

Module 2/3: PsychoSocial Spiritual Cultural Care

On a scale of 1-10, how *confident* are you in your ability to perform each of the following learning objectives?

	Not at all confident — Completely Confident<br>1 2 3 4 5 6 7 8 9 10
Describe the role of the psychological, social, spiritual and cultural domains in palliative care ()	
Screen patients to identify psychosocial, spiritual, and cultural needs ()	
Provide basic support and other resources to address psychosocial, spiritual, and cultural needs identified during screening ()	

Module 4: Serious Illness Communication (Part 1)

Confidence On a scale of 1-10, how *confident* are you in your ability to perform each of the following learning objectives?

	Not at all confident — Completely Confident<br>1 2 3 4 5 6 7 8 9 10
List best practices when communicating with seriously ill patients and families. ()	
Describe four skills to enhance your communication with seriously ill patients and families. ()	

Property of UC Regents, B. Calton, B. Sumser, N. Saks, T. Reid, N. Shepard-Lopez

Module 5: Serious Illness Communication (Part 2)

On a scale of 1-10, how *confident* are you in your ability to perform each of the following learning objectives?

	Not at all confident Completely Confident<br>1 2 3 4 5 6 7 8 9 10
Demonstrate four skills to enhance your communication with seriously ill patients ()	
Describe how capacities impact your communication with seriously ill patients ()	

Module 6: Pain Management

On a scale of 1-10, how *confident* are you in your ability to perform each of the following learning objectives?

	Not at all confident Completely Confident<br>1 2 3 4 5 6 7 8 9 10
Discuss how a patient's biologic/psychosocial/spiritual/cultural identity informs their experience of pain ()	
Identify helpful pain assessment tools ()	
Describe a multi-modal approach to managing pain ()	

Module 7: Symptom Management

On a scale of 1-10, how *confident* are you in your ability to perform each of the following learning objectives?

	Not at all confident Completely Confident<br>1 2 3 4 5 6 7 8 9 10
Appreciate the frequency with which seriously ill patients experience symptoms ()	
Demonstrate a holistic approach to symptom management ()	
Identify key assessment and management strategies for three of the most common symptoms ()	

Module 8: Advance Care Planning

On a scale of 1-10, how *confident* are you in your ability to perform each of the following learning objectives?

	Not at all confident Completely Confident<br>1 2 3 4 5 6 7 8 9 10
Describe the process of ACP ()	
List tools available to assist patients and families in documenting care preferences ()	
Identify communication strategies to introduce and explore a patient's care preferences ()	

Module 9: Care Near the End of Life

On a scale of 1-10, how *confident* are you in your ability to perform each of the following learning objectives?

	Not at all confident Completely Confident<br>1 2 3 4 5 6 7 8 9 10
Describe what patients/families want at the end of life ()	
Identify common signs and symptoms in final days of life ()	
Recognize religious/spiritual/cultural practices and rituals before/after death ()	
Name strategies to identify and address grief and bereavement needs ()	
Recognize approaches to identifying professional grief ()	

Property of UC Regents, B. Calton, B. Sumser, N. Saks, T. Reid, N. Shepard-Lopez

Post-Module Survey(s)

At completion of a module(s), send out the following electronic survey.

Include the confidence questions above on pages 1-4 for the modules you just taught and also include the following questions:

How much do you agree with each of the following statements regarding this module?

	Strongly disagree (-3)	Disagree (-2)	Somewhat disagree (-1)	Somewhat agree (1)	Agree (2)	Strongly agree (3)
The cases and examples used in this module were relevant to my practice	○	○	○	○	○	○
This module's content reflected an interprofessional viewpoint	○	○	○	○	○	○
The presentation of this module was appropriately interactive	○	○	○	○	○	○

The time allotted to this module was...

○ Far too little (-2)

○ Slightly too little (-1)

○ About right (0)

○ Slightly too much (1)

○ Far too much (2)

What were this module's **strengths**? What worked well?

What were this module's **weaknesses**? How could it be improved?

Property of UC Regents, B. Calton, B. Sumser, N. Saks, T. Reid, N. Shepard-Lopez
