# Supplementary material for: An Interprofessional Primary Palliative Care Curriculum for Health Care Trainees and Practicing Clinicians
Source: Palliat Med Rep. 2022 May 5;3(1):80–6. doi: 10.1089/pmr.2021.0074 (PMC9153988; doi:10.1089/pmr.2021.0074)
Supplement: Supplemental data [file Suppl_AppendixSA2.pdf]

Final Evaluation of Curriculum Survey

Send after completion of all nine modules

The overall quality of this course (all modules) was

- ○ Poor
- ○ Adequate
- ○ Good
- ○ Very Good
- ○ Excellent

How useful do you feel what you learned in this course will be to your clinical practice?

- ○ Not at all useful
- ○ Slightly useful
- ○ Moderately useful
- ○ Very useful
- ○ Extremely useful

For Calculation of a Net Promoter Score:

How likely would you be to recommend this course to others interested in palliative care education?

- ○ 0 (Not Likely at All)
- ○ 1
- ○ 2
- ○ 3
- ○ 4
- ○ 5
- ○ 6
- ○ 7
- ○ 8
- ○ 9
- ○ 10 (Extremely Likely)

What are 1-3 things you plan to do differently in your clinical practice as a result of this course? We will email you these as learning reinforcement in one month.

- ◯ Takeaway/plan #1 ______________________________
- ◯ Takeaway/plan #2 ______________________________
- ◯ Takeaway/plan #3 ______________________________

Please share any comments (other than what you may have already provided regarding specific modules) you may have regarding the course as a whole.

____

____

____

____

Property of UC Regents, B. Calton, B. Sumser, N. Saks, T. Reid, N. Shepard-Lopez
