# Supplementary material for: An Interprofessional Primary Palliative Care Curriculum for Health Care Trainees and Practicing Clinicians
Source: Palliat Med Rep. 2022 May 5;3(1):80–6. doi: 10.1089/pmr.2021.0074 (PMC9153988; doi:10.1089/pmr.2021.0074)
Supplement: Supplemental data [file Suppl_Data.pdf]

Full curriculum is available for download and use here:
https://palliativemedicine.ucsf.edu/education/PPCC
